# Supplementary material for: NSC95397 Is a Novel HIV-1 Latency-Reversing Agent
Source: Viruses. 2024 Nov 16;16(11):1783. doi: 10.3390/v16111783 (PMC11599149; doi:10.3390/v16111783)
Supplement: Supplementary file 1 [file viruses-16-01783-s001.zip › viruses-3286261-supplementary.pdf]

Supplement File S1

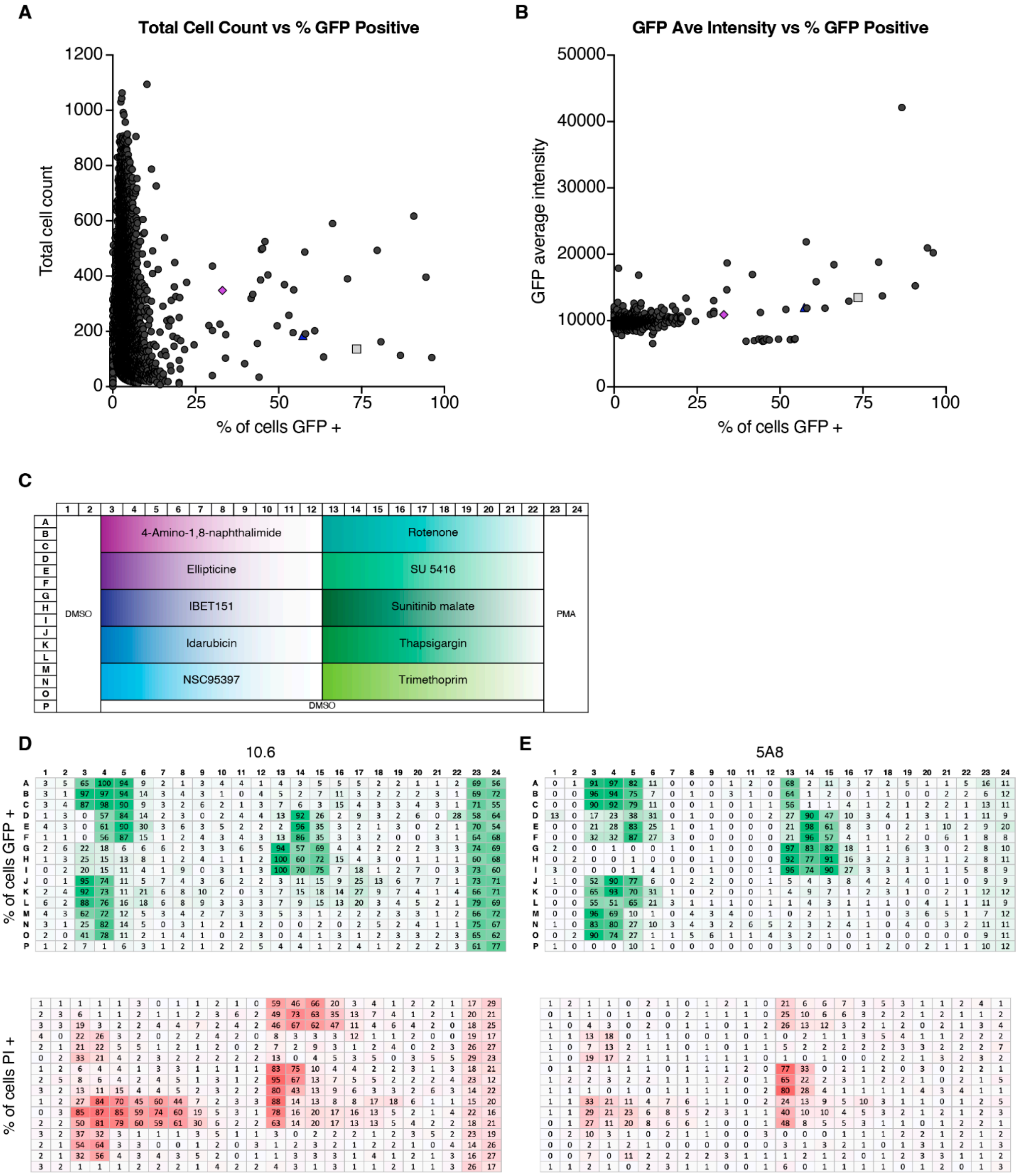

Supplemental Figure S1. NSC95397 and IBET151 were identified as HIV-1 LRAs of interest from a medium-throughput screen. A-B) The raw screen data of total cell count or GFP intensity vs percent of cells GFP positive. The grey square and purple diamond are two known latency reversal agents, PMA and IBET151, respectively, and the blue triangle is a previously unknown LRA, NSC95397. **C)** Example plate map of the EC_{50} screen. Each compound was screened in triplicate and across the plate is a 4-fold concentration decrease. The first two columns and last row were treated with the vehicle control, DMSO, and the last two columns were treated with the positive control, PMA. **D-E)** The percent of GFP positive cells, top green plate, and dead PI positive cell, lower red plate, measured during our EC_{50} titration experiment in J-Lat 10.6 (**D**) and 5A8 cells (**E**). Related to Figure 1.

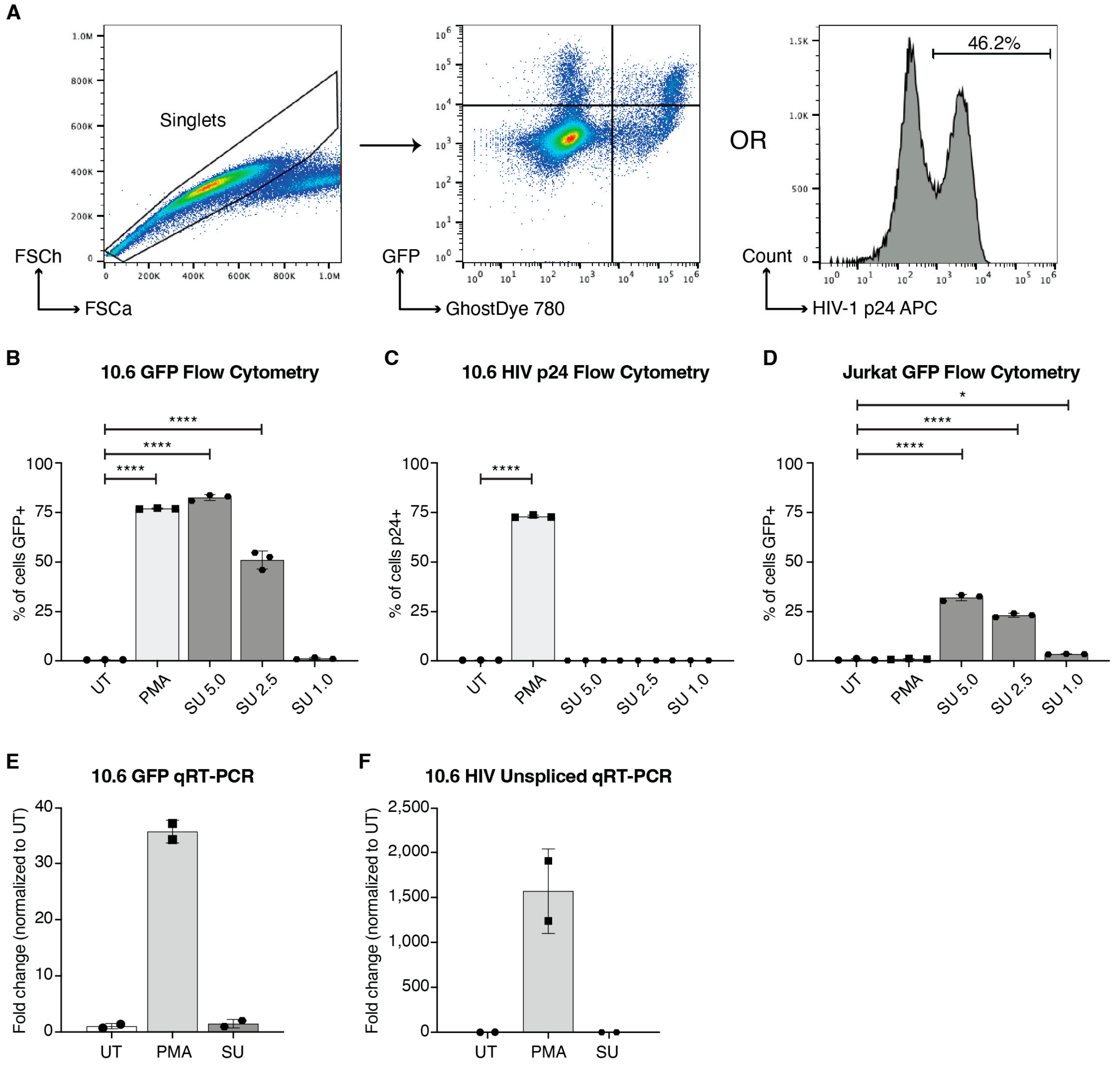

Supplemental Figure S2. Sunitinib is one of a subset of false positives identified in a J-Lat screen for LRAs. A) Gating strategy used for all flow cytometry analysis. J-Lat 10.6 or parental Jurkat cells were untreated or treated with 5.0 ng/mL PMA (positive control), or a titration of sunitinib (SU) from 5.0 to 1.0 μM. **A-B)** Bar graphs showing GFP (**A**) or intracellular HIV-1 p24 (**B**) measured via flow cytometry in J-Lat 10.6 cells. **C)** A bar graph of GFP measured via flow cytometry in treated Jurkat cells. **D-E)** Bar graphs showing GFP (**D**) or unspliced HIV-1 RNA transcripts (**E**) measured via qRT-PCR. Related to Figure 2.

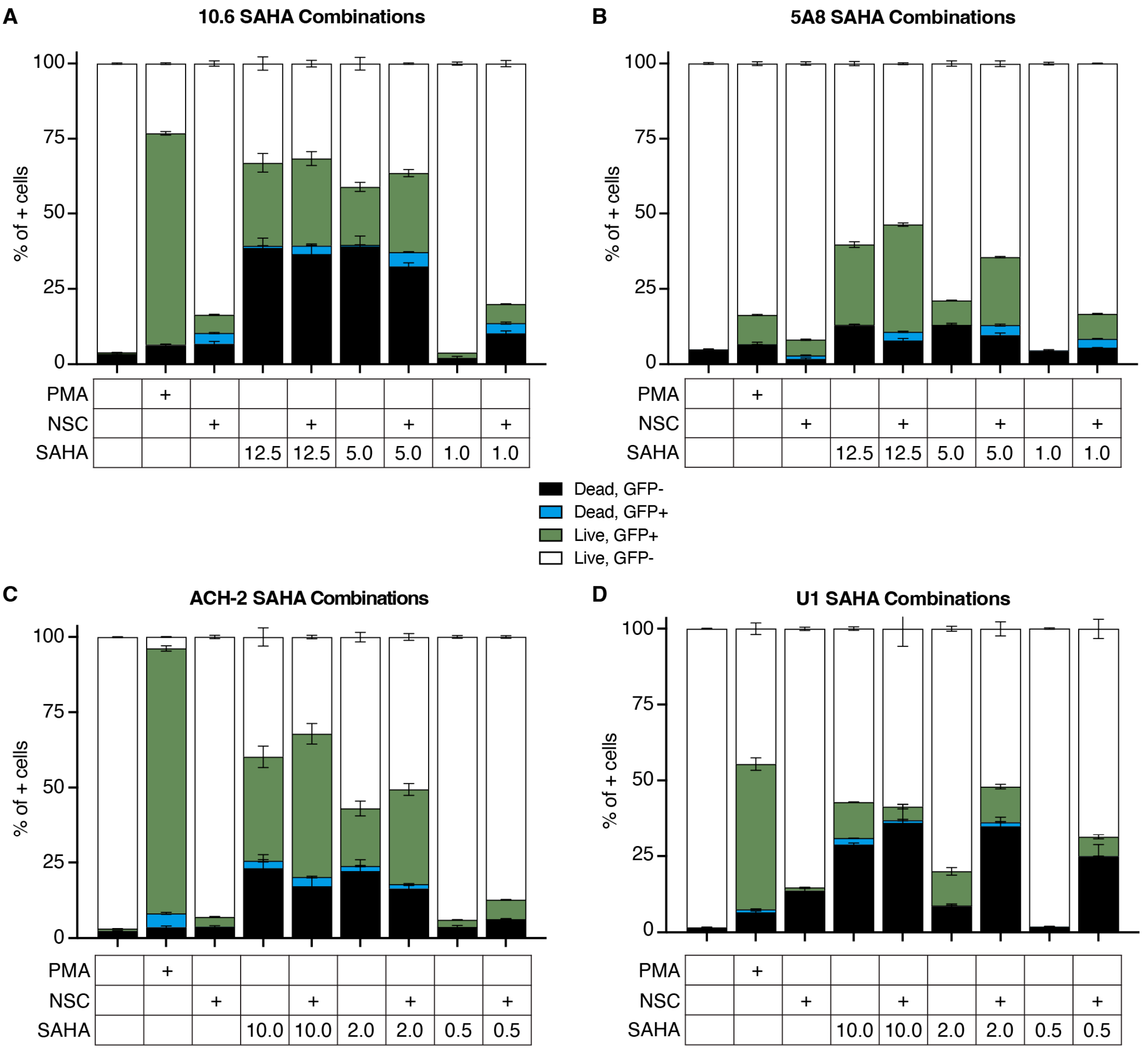

Supplemental Figure S3. NSC95397 increases HIV-1 reactivation and cytotoxicity in combination with SAHA. Stacked graphs show the percentage of cells from the full population that are either dead (ghost dye positive) and GFP- (black), dead and GFP+ (blue), live and GFP+ (green), or live and GFP- (white) stacked to a total of 100%. **A-B)** J-Lat 10.6 (**A**) or 5A8 (**B**) cells were untreated or treated with 5.0 ng/mL PMA, 1.5 µM NSC95397 (NSC), a titration of SAHA from 12.5 to 1.0 µM, or a titration of SAHA with NSC held constant at 1.5 µM. **C-D)** ACH-2 (**C**) or U1 (**D**) cells were untreated or treated with 5.0 ng/mL (**C**) or 20.0 ng/mL PMA (**D**), 2.5 µM NSC,

a titration of SAHA from 10.0 to 0.5 μM, or a titration of SAHA with NSC held constant at 2.5 μM. Related to Figure 3.

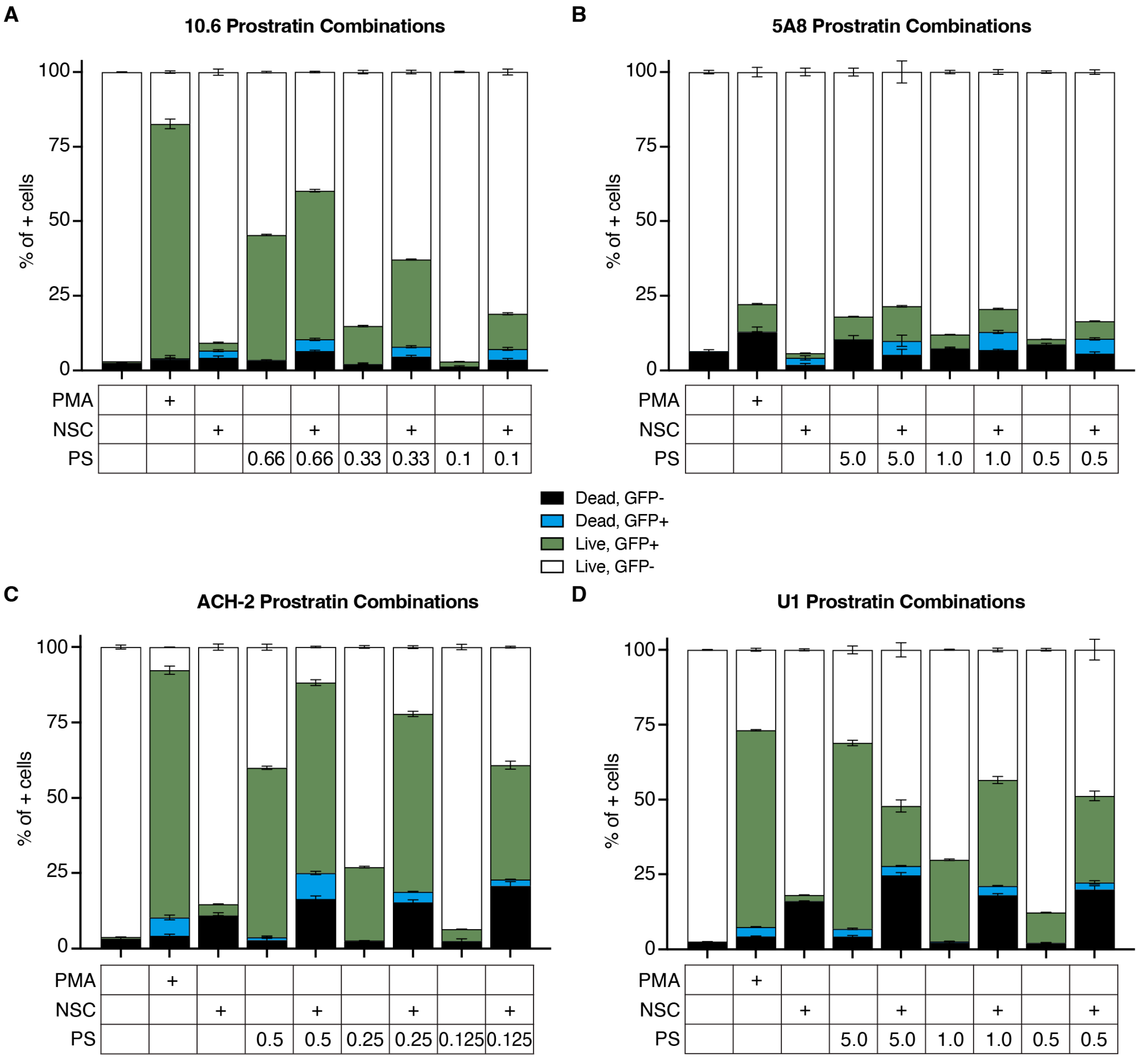

Supplemental Figure S4. NSC95397 increases HIV-1 reactivation and cytotoxicity in combination with prostratin. Stacked graphs show the percentage of cells from the full population that are either dead (ghost dye positive) and GFP- (black), dead and GFP+ (blue), live and GFP+ (green), or live and GFP- (white) stacked to a total of 100%. **A-B)** J-Lat 10.6 (**A**) or 5A8 (**B**) cells were untreated or treated with 5.0 ng/mL PMA, NSC95397 (NSC) at 1.5μM, a titration of prostratin (PS) from 0.66 to 0.1 μM (**A**) or 5.0 to 0.5 μM (**B**), or a titration of PS with NSC held constant at 1.5 μM. **C-D)** ACH-2 (**C**) or U1 (**D**) cells were untreated or treated with 5.0

ng/mL (**C**) or 20.0 ng/mL PMA (**D**), 2.5 μM NSC, a titration of PS from 0.5 to 0.125 μM (**C**) or 5.0 to 0.5 μM (**D**), or a titration of PS with NSC held constant at 2.5 μM. Related to Figure 4.

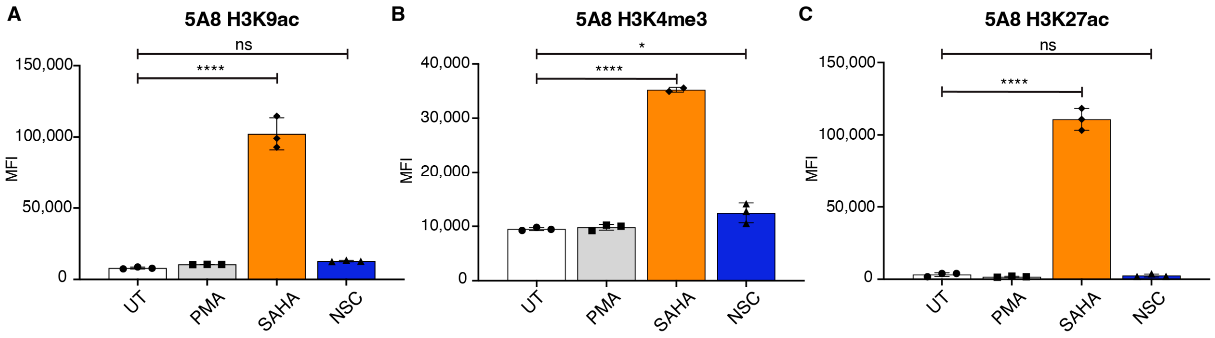

Supplemental Figure S5. NSC95397 does not change global histone modifications for open chromatin. A-C) J-Lat 5A8 cells were untreated or treated with 5.0 ng/mL PMA, 25.0 μM SAHA (positive control), or NSC95397 (NSC) at 2.5 μM. Bar graphs show H3K9ac (**A**), H3K4me3 (**B**), or H3K27ac (**C**) MFI measured by flow cytometry. Bar graphs show GFP MFI measured via flow cytometry. Results were analyzed with a one-way ANOVA with Turkey's multiple comparisons tests; ns = not significant, * = $p \leq 0.0332$, ** = $p \leq 0.0021$, *** = $p \leq 0.0002$, **** = $p \leq 0.0001$. Related to Figure 5.

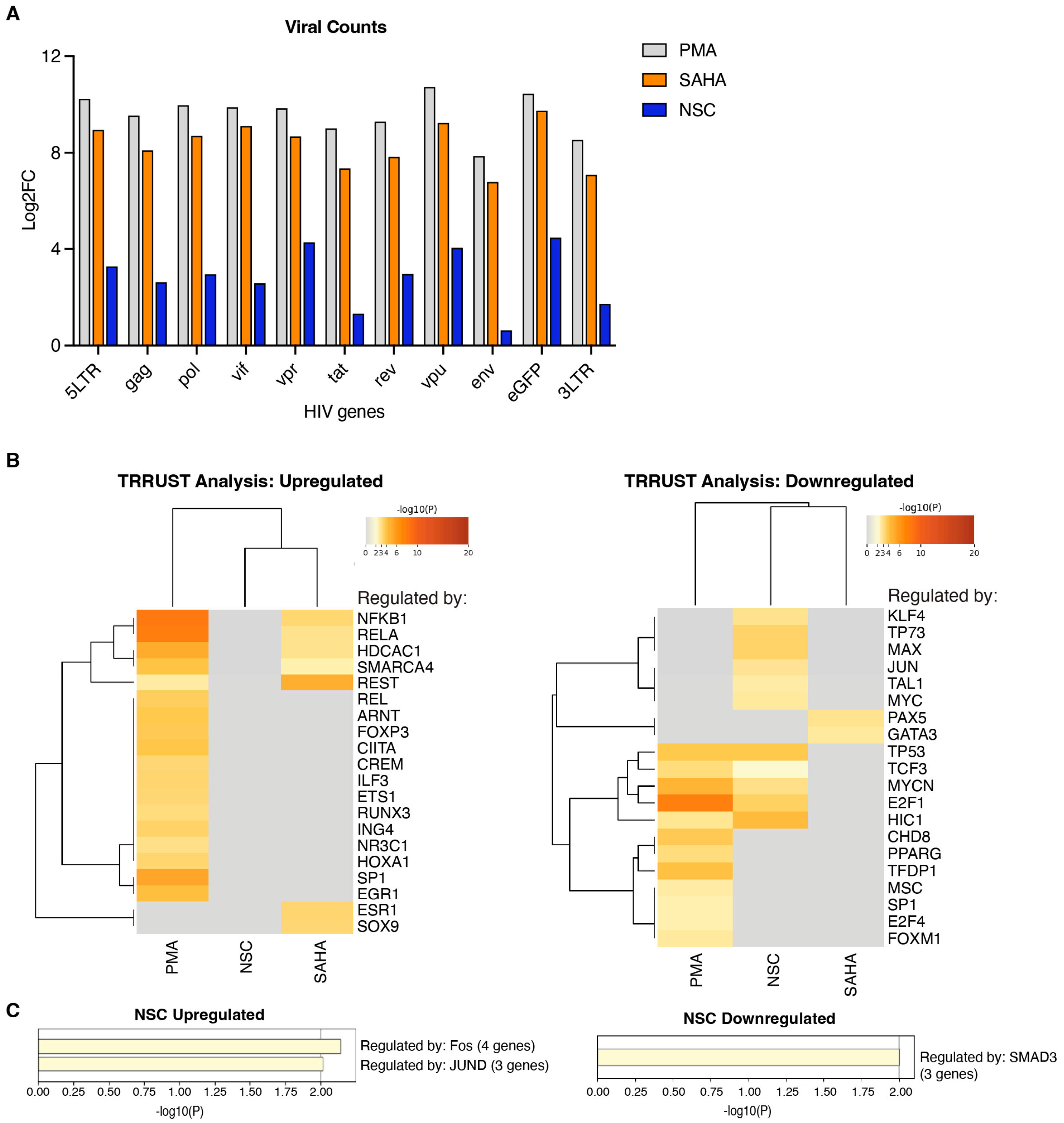

Supplemental Figure S6. NSC95397 has a minimal effect on global transcription. J-Lat 10.6 cells were untreated or treated with 5.0 ng/mL PMA, 25.0 µM SAHA, or 5.0 µM NSC95397 (NSC) and used for bulk RNA sequencing in four biological replicates. **A)** Bar graph showing log2 fold change of HIV-1 associated reads normalizing cells treated with PMA (grey), SAHA (orange), or NSC (blue) against untreated cells. **B-C)** Gene ontology using Metascape for TRRUST analysis

of upregulated (left) and downregulated (right) genes for all three treatments (**B**) and NSC alone (**C**). Related to Figure 6 and Supplemental Figure S7.

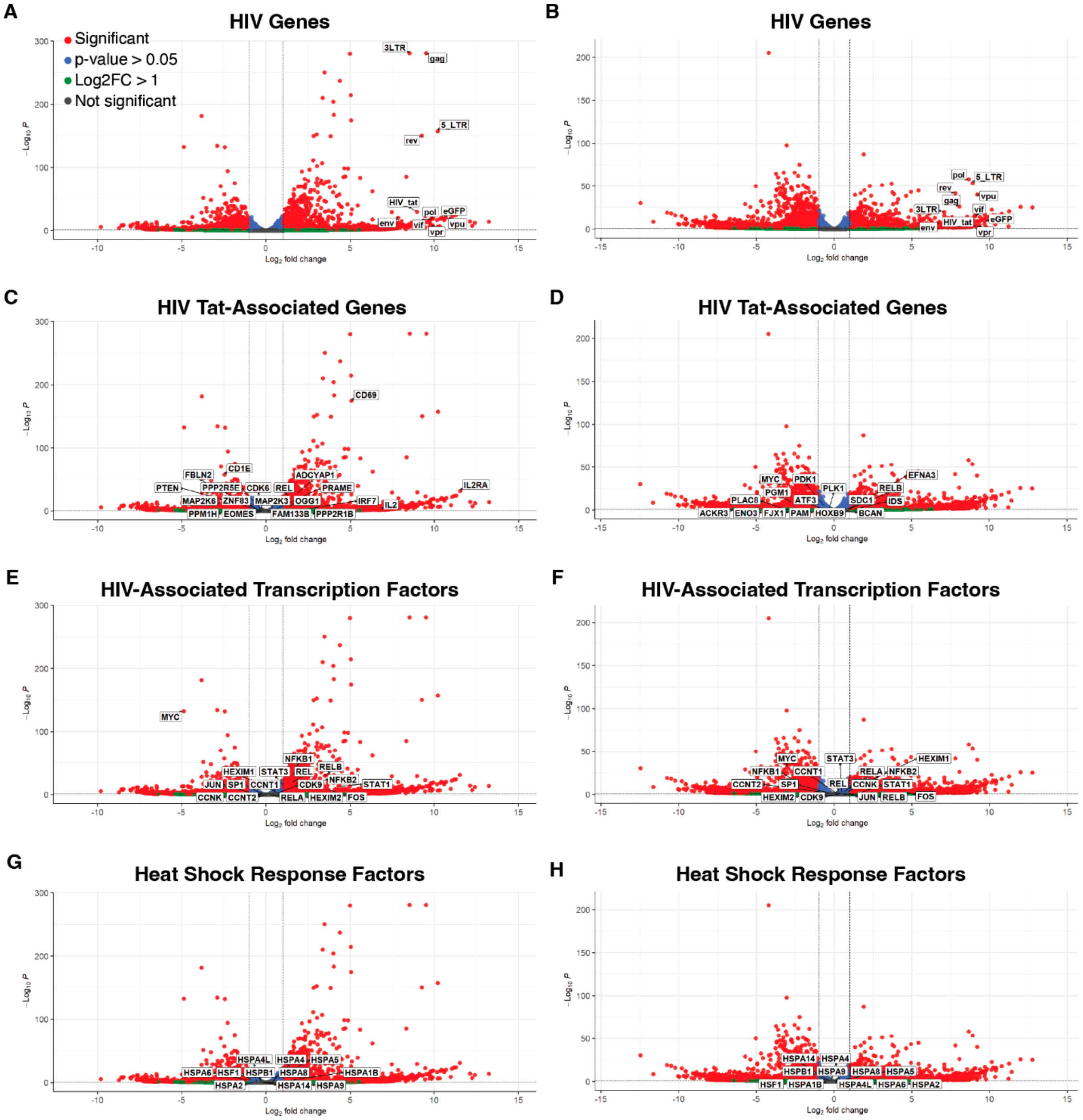

Supplemental Figure S7. PMA and SAHA change latency modulating pathways. J-Lat 10.6 cells were untreated or treated with 5.0 ng/mL PMA or 25.0 μM SAHA and used for bulk RNA sequencing in four biological replicates. **A-B)** Volcano plot highlighting HIV-associated reads in cells treated with PMA (**A**) or SAHA (**B**). **C-D)** Volcano plots highlighting HIV-associated transcription factors in cells treated with PMA (**C**) or SAHA (**D**). **E-F)** Volcano plots highlighting selected HIV Tat-modulated genes in cells treated with PMA (**E**) or SAHA (**F**). **G-H)** Volcano plots

highlighting key heat shock response genes in cells treated with PMA (**G**) or SAHA (**H**). Cut-offs are drawn for log2 fold change above 1 and p-value greater than 0.05 where reads are separated to non-significant and non-enriched (gray), non-significant with log2 fold change above 1.0 (green), significant and log2 fold change below 1.0 (blue), or significant and log2 fold change above 1.0 (red). Related to Figure 6 and Supplemental Figure S6.

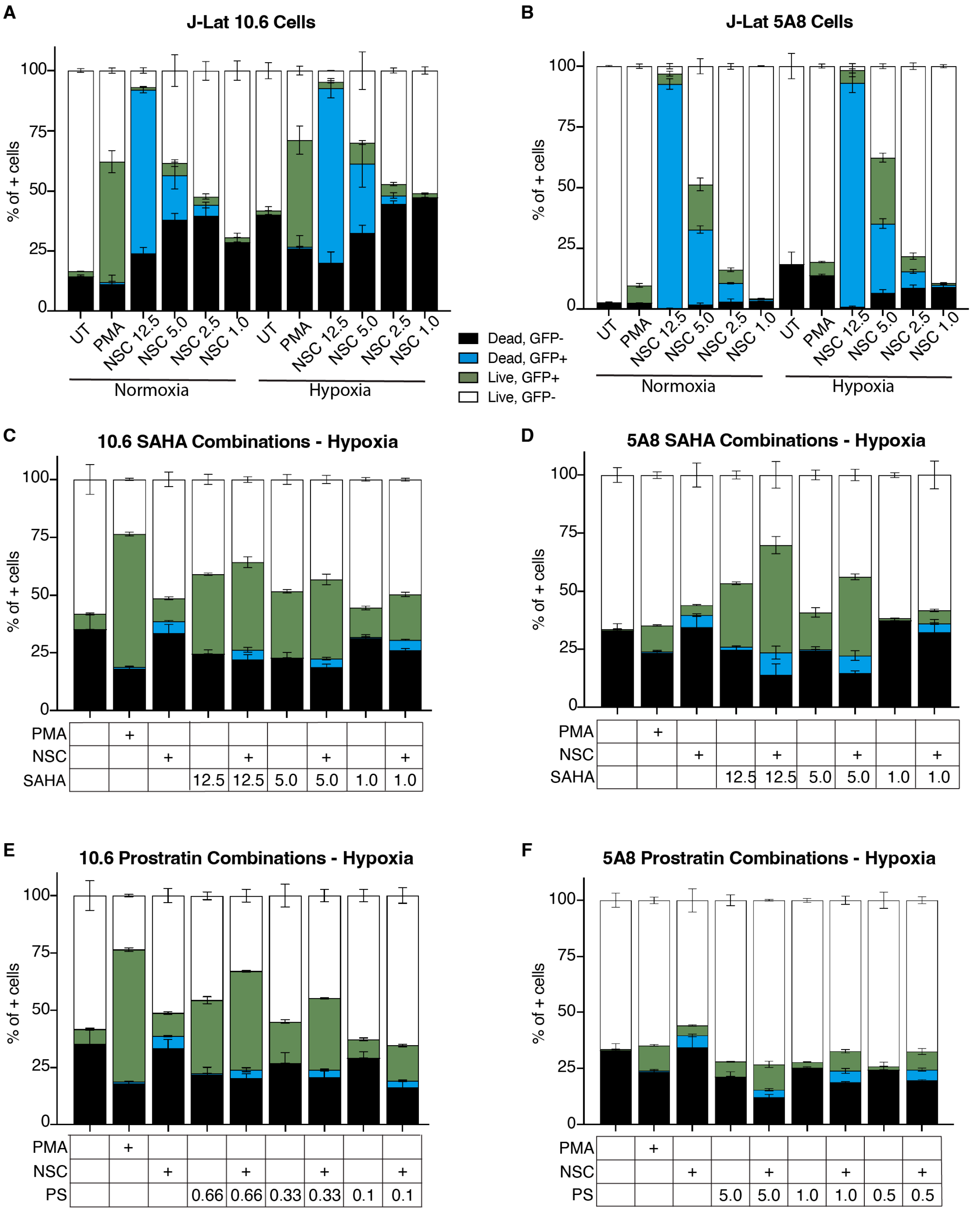

Supplemental Figure S8. NSC95397 alone and in combination with LRAs increases HIV reactivation and cytotoxicity in cells under hypoxia. A-F) Stacked graphs show the

percentage of cells from the full population that are either dead (ghost dye positive) and GFP- (black), dead and GFP+ (blue), live and GFP+ (green), or live and GFP- (white) stacked to a total of 100%. Bar graphs show the percent of cells that are GFP+ from the full population. **A-B)** J-Lat 10.6 (**A**) or 5A8 (**B**) cells were untreated or treated with 5.0 ng/mL PMA (positive control), or a titration of NSC95397 (NSC) from 5 μM to 1.0 μM **C-D)** J-Lat 10.6 (**C**) or 5A8 (**D**) cells were untreated or treated with 5.0 ng/mL PMA (positive control), NSC95397 (NSC) at 1.5 μM, a titration of SAHA from 12.5 to 1.0 μM, or a titration of SAHA with NSC held constant at 1.5 μM. **E-F)** J-Lat 10.6 (**E**) or 5A8 (**F**) cells were untreated or treated with 5.0 ng/mL PMA, NSC at 1.5 μM, a titration of prostratin (PS) from 0.66 to 0.1 μM (**E**) or 5.0 to 0.5 μM (**F**), or a titration of PS with NSC held constant at 1.5 μM. Results were analyzed with a one-way ANOVA with Turkey's multiple comparisons tests; ns = not significant, * = $p \leq 0.0332$, ** = $p \leq 0.0021$, *** = $p \leq 0.0002$, **** = $p \leq 0.0001$. Related to Figure 6 and Supplemental Figure S7.

Table 1

Cell Line	NSC95397 [uM]	SAHA [uM]	CI	Drug-Drug Effect
J-Lat 10.6	1.5	12.5	1.22	Moderate Antagonism
	1.5	5.0	0.85	Slight Synergism
	1.5	1.0	0.89	Slight Synergism
J-Lat 5A8	1.5	12.5	1.13	Slight Antagonism
	1.5	5.0	0.87	Slight Synergism
	1.5	1.0	0.91	Nearly Additive
ACH-2	2.5	10.0	0.75	Moderate Synergism
	2.5	2.0	0.50	Synergism
	2.5	0.5	0.88	Slight Synergism
U1	2.5	10.0	1.49	Antagonism
	2.5	2.0	0.18	Strong Synergism
	2.5	0.5	0.10	Strong Synergism
J-Lat 10.6 (Hypoxia)	1.5	12.5	0.88	Slight Synergism
	1.5	5.0	0.67	Synergism
	1.5	1.0	0.67	Synergism
J-Lat 5A8 (Hypoxia)	1.5	12.5	1.01	Nearly Additive
	1.5	5.0	0.79	Moderate Synergism
	1.5	1.0	1.06	Nearly Additive

Table S1. Summary of NSC95397 and SAHA combination index synergy calculations. CI (combination index) values for drug-drug effect were calculated using CompuSyn. Concentrations with CI ≤ 0.90 are considered to be synergistic (green)- or greater than additive (with lower values indicating stronger synergy). Concentrations close to 1.0 are considered to have an additive effect (white), in which the two drugs exhibit the same effect from an independent dosage. Concentrations with CI ≥ 1.10 are considered to be antagonistic (purple), or less than additive (with higher values indicating stronger antagonism). Unless otherwise stated, experiments were done in normoxic conditions.

Table 2

Cell Line	NSC95397 [uM]	Prostratin [uM]	CI	Drug-Drug Effect
J-Lat 10.6	1.5	0.660	1.00	Near Additive
	1.5	0.330	0.84	Moderate Synergism
	1.5	0.100	0.62	Synergism
J-Lat 5A8	1.5	5.000	2.63	Antagonism
	1.5	1.000	1.05	Near Additive
	1.5	0.500	0.92	Near Additive
ACH-2	2.5	0.500	1.00	Near Additive
	2.5	0.250	0.76	Moderate Synergism
	2.5	0.125	0.65	Synergism
U1	2.5	5.000	2.99	Antagonism
	2.5	1.000	0.44	Synergism
	2.5	0.500	0.30	Synergism
J-Lat 10.6 (Hypoxia)	1.5	0.660	1.01	Near Additive
	1.5	0.330	0.84	Moderate Synergism
	1.5	0.100	0.85	Slight Synergism
J-Lat 5A8 (Hypoxia)	1.5	5.000	1.07	Near Additive
	1.5	1.000	0.81	Moderate Synergism
	1.5	0.500	0.79	Moderate Synergism

Table S2. Summary of NSC95397 and prostratin combination index synergy calculations. CI (combination index) values for drug-drug effect were calculated using CompuSyn. Concentrations with CI ≤ 0.90 are considered to be synergistic (green), or greater than additive (with lower values indicating stronger synergy). Concentrations close to 1.0 are considered to have an additive effect (white), in which the two drugs exhibit the same effect from an independent dosage. Concentrations with CI ≥ 1.10 are considered to be antagonistic (purple), or less than additive (with higher values indicating stronger antagonism). Unless otherwise stated, experiments were done in normoxic conditions.
